# Cell-free therapy based on extracellular vesicles: a promising therapeutic strategy for peripheral nerve injury

**DOI:** 10.1186/s13287-023-03467-5

**Published:** 2023-09-19

**Authors:** Mojdeh Salehi Namini, Fatemeh Daneshimehr, Nima Beheshtizadeh, Vahid Mansouri, Jafar Ai, Hossein Kargar Jahromi, Somayeh Ebrahimi-Barough

**Affiliations:** 1 Department of Tissue Engineering, School of Advanced Technologies in Medicine, Tehran University of Medical Sciences, Tehran, Iran; 2 Regenerative Medicine Group (REMED), Universal Scientific Education and Research Network (USERN), Tehran, Iran; 3 Digestive Disease Research Institute, Shariati Hospital, Tehran University of Medical Sciences, Tehran, Iran; 4 Research Center for Noncommunicable Diseases, Jahrom University of Medical Sciences, Jahrom, Iran

**Keywords:** Peripheral neve injury, Nerve regeneration, Cell-free-based treatment, Mesenchymal stromal cells, Extracellular vesicle, Exosomes

## Abstract

Peripheral nerve injury (PNI) is one of the public health concerns that can result in a loss of sensory or motor function in the areas in which injured and non-injured nerves come together. Up until now, there has been no optimized therapy for complete nerve regeneration after PNI. Exosome-based therapies are an emerging and effective therapeutic strategy for promoting nerve regeneration and functional recovery. Exosomes, as natural extracellular vesicles, contain bioactive molecules for intracellular communications and nervous tissue function, which could overcome the challenges of cell-based therapies. Furthermore, the bioactivity and ability of exosomes to deliver various types of agents, such as proteins and microRNA, have made exosomes a potential approach for neurotherapeutics. However, the type of cell origin, dosage, and targeted delivery of exosomes still pose challenges for the clinical translation of exosome therapeutics. In this review, we have focused on Schwann cell and mesenchymal stem cell (MSC)-derived exosomes in nerve tissue regeneration. Also, we expressed the current understanding of MSC-derived exosomes related to nerve regeneration and provided insights for developing a cell-free MSC therapeutic strategy for nerve injury.

## Introduction

Peripheral nerve injury (PNI) is a common neurological disorder in the clinic that seriously influences human health [1]. In these cases, patients endure neuropathic pain, which can result in dysfunction of the sensory and motor systems and also cause disability [2, 3]. Hence, the development of novel treatment strategies to enhance peripheral nerve repair post-injury, especially those that can accelerate axonal nerve regeneration, is necessary [4].

Several factors are involved in axonal outgrowth in peripheral nerve regeneration, such as the transformation of the phenotype of Schwann cells (SCs), the infiltration of immune cells, neurovascular regeneration, and also neuronal soma formation, which plays a key role in the initiation and control of axonal regeneration [5, 6]. On the other hand, one of the main challenges in the regeneration of peripheral nerves is the low speed of axon growth (only 1 mm per day) [7]. Although the peripheral nerve has the potential for self-regeneration, in several cases, such as the long length of the nerve defect, the long duration between injury and treatment limits its spontaneous self-regeneration [8]. Also, self-regeneration is often inadequate and might be prevented by scar formation [8]. One of the strategies in PNI is direct neurorrhaphy, but it can be applied only in cases with short gaps [9]. Another strategy for the treatment of large nerve defects is autologous nerve grafting, which has served as the gold standard [10]. However, autologous nerve grafts via microsurgical procedure are limited due to insufficient nerve sources, potential donor site dysfunction, size mismatch between donor nerves and nerve grafts, and other complications [11, 12].

In the field of peripheral nerve injury (PNI), several studies have been done in the past few years to find a way to replace the autologous nerve graft method in clinics with a new way to speed up axonal regeneration without harming other nerves [13]. One of the new therapeutic strategies in regenerative medicine is cell therapy. Although cell therapy has demonstrated beneficial effects on peripheral nerve tissue treatment and regeneration, it still has several drawbacks, such as a decreased survival rate of the engrafted cells, the low regenerative capacity of cells, tumorigenesis, immune-mediated rejection, the risk of capillary blockade during infusion, and ethical concerns that hinder the wide use of cells in the clinic [14, 15]. One of the alternative therapeutic strategies is the design of various tissue-engineered nerve guide conduits (NGCs) to provide mechanical, biological, and biochemical supports [16]. Although these synthetic NGCs with or without cells and growth factors (GFs) have been shown to be beneficial, the results of their use in the treatment of PNI remain far from ideal [17]. Exosome-based therapy with or without NGCs is now used in PNI as an alternative to cell therapy and tissue-engineered NGC alone.

## Exosomes as extracellular vesicles

Johnstone et al. [18] showed in 1970 that exosomes are extracellular vesicles (EV) that carry different substrates. After that, several extracellular vehicles have been identified and can now be classified as apoptotic bodies, microvesicles (MVs), and exosomes (EXOs), depending on their biogenesis and size. According to this classification, EXOs are the smallest extracellular vesicles (30–100 nm) with a lipid bilayer membrane released by all types of cells, such as Schwann cells (SCs) and mesenchymal stromal cells (MSCs) [19]. Furthermore, EXOs are the key mediators of paracrine mechanisms, and the biogenesis of EXOs is the endocytic pathway. Recently, several studies have focused on EXOs, and they have demonstrated that different types of EXOs are released from certain types of cells that are associated with pathological and physiological conditions, such as neurodegenerative diseases, tumors, and tissue fibrosis [20]. On the other hand, EXOs have several cellular signaling molecules like DNA, proteins, lipids, mRNA, miRNA, lncRNA, and circRNA that mediate intercellular communication due to transferring these types of cargo [21–23].

### The therapeutic effects of Schwann cells-derived exosome on PNI

Schwan cells(SCs), the glial cells of the PNS, are a critical factor for maintaining homeostasis in the nerves and facilitating the regeneration process of the PNI [24]. SCs provide the nutrition to support axonal regeneration, and SCs are the basic cell type that organizes the formation of myelin sheaths along the axon [25]. A chain of molecular and cellular reactions known as Wallerian degeneration (WD) was initiated in PNI. In this case, the peripheral glia (the SCs) were dedifferentiated into a non-myelinating cell type and proliferated to omit the endoneurial myelin and all debris [26–28]. Also, SCs secrete neurotrophic factors and specific cytokines [27]. Furthermore, miRNAs can be conveyed by EXOs from SCs to neurons to promote the regeneration of PNI. Indeed, the level of miRNA expression by SCs plays the main role in the nerve regeneration process [29, 30].

The results of several studies have demonstrated that miRNA can augment SC proliferation and axon myelination during development and injury [31]. SC-derived exosomes have been shown to be internalized by axons and enhance neurite outgrowth, and direct injection of SC-derived exosomes can improve axon growth following in vivo PNI [32]. Moreover, SC-derived exosomes can change the growth cone phenotype to a pro-regeneration morphology and decrease the activity of the GTPase RhoA, which plays a role in axon retraction and collapse of the growth cone [32].

Altogether, these studies have demonstrated that SCs can release EXOs, and these SC-derived exosomes have been illustrated to play an essential role in neurodegeneration, neurodevelopment, and neuroprotection [33, 34]. Also, a study illustrated that SC-derived exosomes can increase the axonal regeneration rate of dorsal root ganglion neurons in in vitro and in vivo investigations, indicating the role of SC-derived exosomes in axonal regeneration [32]. On the other hand, these promotive effects are dependent on the type of SC-derived exosomes released from various phenotypic SCs [35].

**Fig. 1:**
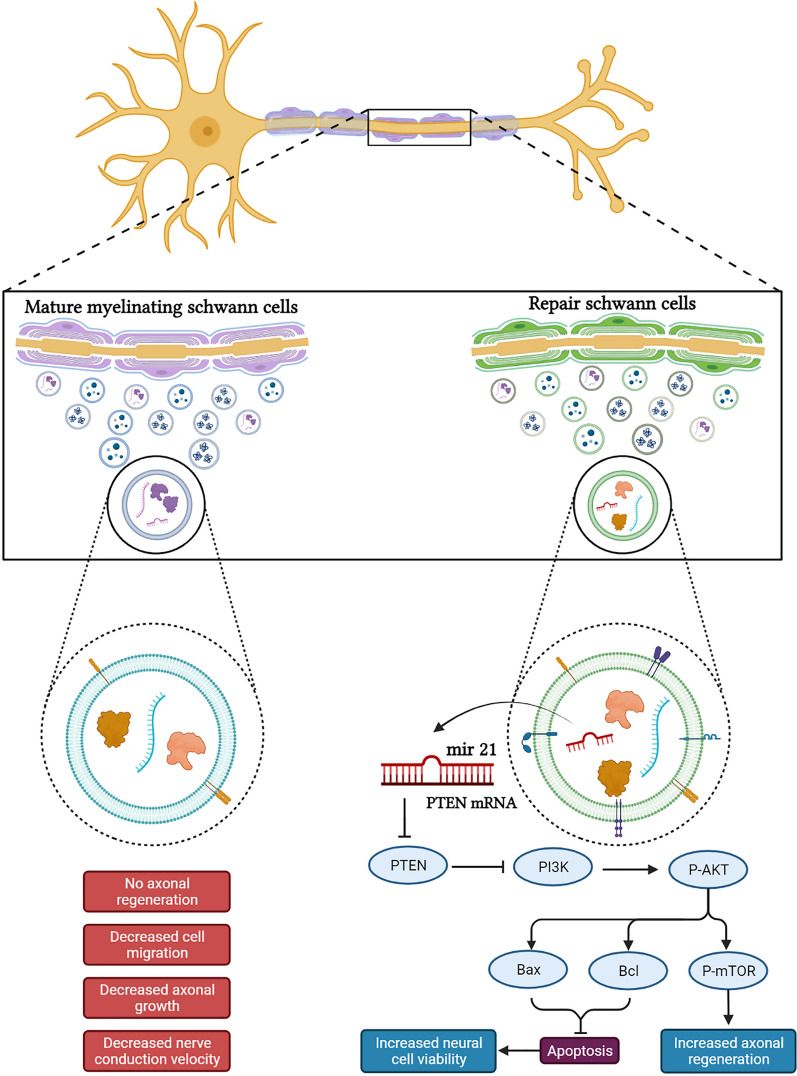
Illustrating exosome contents that promote axon regeneration through the PI3/AKT signaling pathway. Schwann cell-derived exosomes released from different phenotypic Schwann cells, like mature SCs and repair SCs, carry different cargoes that influence their functions. For example, repair SC-derived exosomes exhibit axonal regeneration after nerve injury due to their containing miRNA-21. miRNA-21 can cause the downregulation of phosphatase and tensin homolog (PTEN) and consequently the activation of phosphoinositide 3-kinase (PI3K) in the neurons. Also, SC-derived exosomes can inhibit neuron apoptosis and increase cell viability. On the other hand, exosomes derived from mature myelinating SCs cannot promote axonal regeneration and also inhibit SC migration

Recently, Lopez-Leal et al. [35] demonstrated that only the SC-derived exosomes secreted by repair SCs enhanced axonal regeneration after PNI, but they did not show these promotive effects in the differentiated SCs. Indeed, repair of SC-derived exosomes mediated the effect of promoting neurite growth from dorsal root ganglia explants by downregulating PTEN, activating PI3K pathways, and also transferring exosomal miRNA-21 (Fig. 1) [36].

According to the results of this study, it is obvious that the repair of SC-derived exosomes shuttled specific proteins and miRNAs that enhanced axonal regeneration and the survival of neurons, while the EXOs from differentiated SCs did not exhibit the promotive effect to enhance axonal regeneration due to the number of miRNAs that suppressed cell migration, such as miRNA-21 and miRNA-92a-3p miRNAs, so they could focus on myelination (the main function of differentiated SCs) (Fig. 1) [37]. The positive effects of SC-derived exosomes in the nerve studies are summarized in Table 1.

**Table 1:** Summary of the positive effects of SC-derived exosomes in the nerve injury studies

Effect	Exosome isolation methods	Exosome concentration and duration for treatment	In vitro/In vivo	Signaling pathway/related exosomal cargo	References
SC-derived exosomes increase axonal regeneration and enhance regeneration after sciatic nerve injury	Differential ultracentrifugation	3 μg per DRG for 4 days (in vitro); 2 μg per ml daily for 4 days (in vivo*)*	In vitro and in vivo	SC-derived exosomes change the growth cone to a pro-regenerating morphology and decrease the activity of the GTPase RhoA, involved in growth cone collapse and axon retraction	[32]
Only exosomes from repair SCs enhance axonal regeneration by transferring miRNA-21, but not exosomes from differentiated SCs	Differential ultracentrifugation	120 ng/ml daily for 3 days	In vitro and in vivo	SC reprogramming is dependent on the repair SCs expression of c-Jun and Sox2. Also, expression of miRNA-21 is responsible for the pro-regenerative capacity of repair SCs exosomes, which is associated to PTEN downregulation and PI3-kinase activation in neurons	[35]
Exosomes derived from skin precursor-derived Schwann cells (SKP-SCs) improve neurite outgrowth by activating PI3K/Akt/mTOR/p70S6K and inhibit apoptosis by reversing Bax/Bcl-2 ratio	exoRNeasy Maxi kit	1.0 × 10^8^, 2.0 × 10^8^, 4.0 × 10^8^ for 5 days	In vitro	Exosomes derived from SKP-SCs can activate PI3K/Akt, mTOR, and p70S6k, as well as reduce the Bax/Bcl-2 signaling pathways Also, exosome derived from SKP-SCs can deliver miR-21-5p into sensory neurons	[43]
EVs from skin precursor-derived Schwann cells (SKP-SC-EVs) promote axonal regeneration on injured motor neurons by activating Akt/mTOR/p70S6K	exoRNeasy Maxi kit	0.5 × 10^8^, 1.0 × 10^8^, 2.0 × 10^8^, 4.0 × 10^8^ for 12, 24, 36 h	In vitro and in vivo	SKP-SC-EVs regulate the cell growth and death signaling pathway by Akt/mTOR/p70S6K	[44]
Exosomes derived from differentiated Schwann cells suppressed SCs migration, while exosomes from undifferentiated SCs did not	miRNA exosome kit	Not mentioned about number of exosomes	In vitro	Exosomes released from differentiated SCs regulated SCs migration by modifying in miRNA expression	[45]
Promote outgrowth and myelination of axons and enhance the recovery of motor, sensory and electrophysiological functions of rats	exoRNeasy Maxi kit	4.0 × 10^8^ for 1 day (in vitro); 2.0 × 10^10^ for 12 weeks (in vivo)	In vitro and in vivo	Prevention of motor neurons cell death by Schwann cell-derived exosomes was obtained through blocking the caspase-3 cell death pathway	[46]

Therefore, it can be concluded that various phenotypes of SCs can transfer different exosomal cargoes that are required for specific functions. On the other hand, several studies have reported that SCs can promote cancer growth and dissemination in pancreatic cancer and melanoma. In these cases, tumor cells exploit SCs due to their exosomal cargo, which promotes higher proliferation and inhibits apoptosis [38–41]. As regards obtaining SC-derived exosomes, it is necessary to sacrifice normal nerve tissue to harvest SCs. This disadvantage is the main challenge for clinical translation [42]. Hence, we need further investigation to identify optimal EXO content and an efficient strategy to obtain SC-derived exosomes without the need to sacrifice normal nerve tissue or find an alternative cell type with similar efficiency to SCs [42].

### The therapeutic effects of mesenchymal stromal cells-derived exosome on PNI

**Fig. 2:**
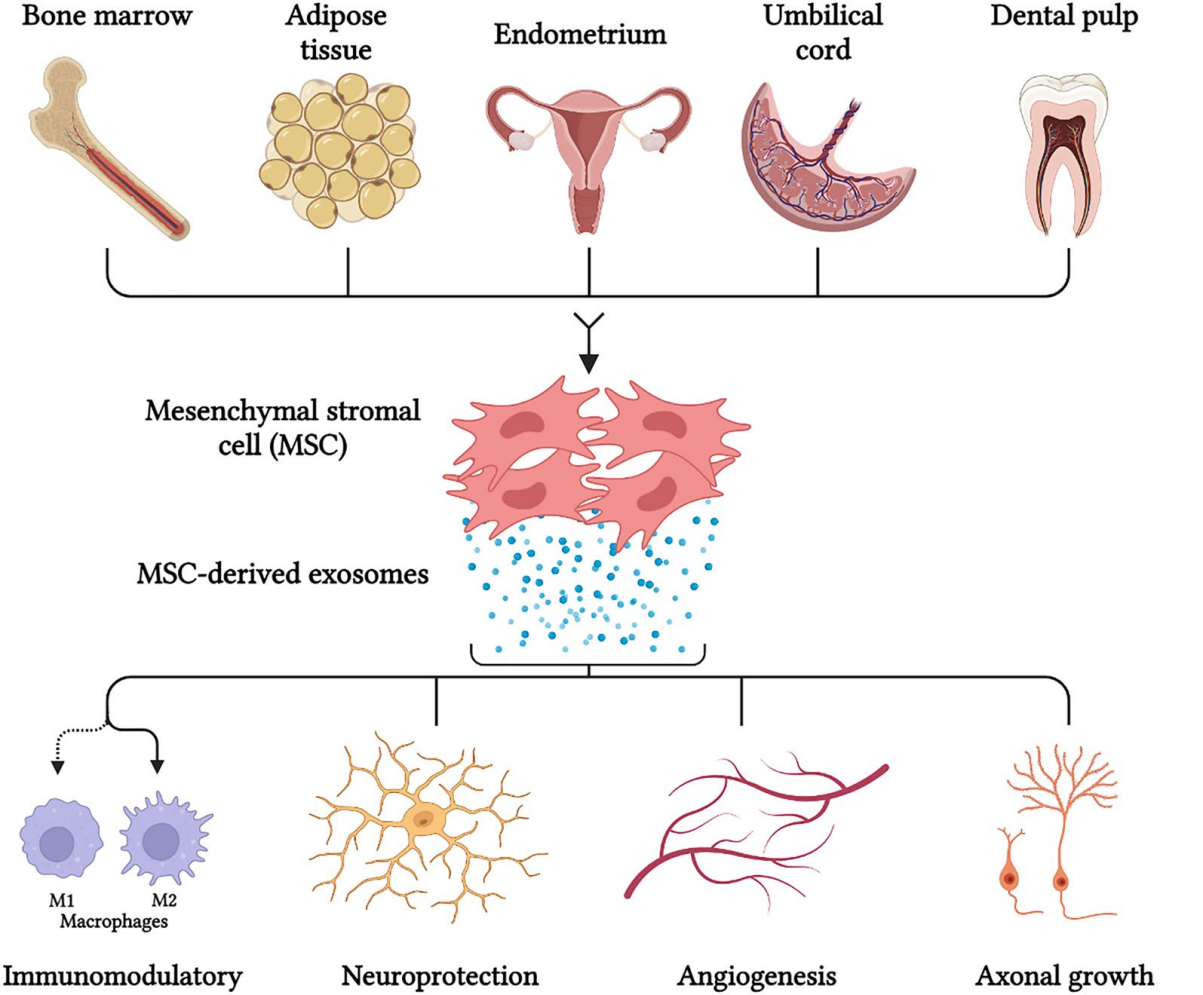
Novel strategies of MSC-derived exosomes for curing nerve injury. MSCs can be isolated from bone marrow, adipose tissue, endometrium tissues, the umbilical cord, and the dental pulp. Their exosomes can regulate nerve-related cellular functions. MSC-derived exosomes are able to modulate neuroinflammation and immune cell reactions, neuroprotection, angiogenesis, and axonal regrowth and remyelination

MSCs are adult multipotent stem cells that can be isolated from various human tissues (i.e., adipose, bone marrow, umbilical cord blood, dental pulp, etc.) [42]. MSCs have been identified as having multi-directional differentiation potential, high self-renewal ability, and low immunogenicity, so they are one of the most common potential off-the-shelf stem cells in cell therapy (Fig. 2) [47].

Several studies have shown that adult multipotent MSCs, which are similar to SCs, can help with functional recovery after PNI by encouraging the growth and survival of neurons. However, there are several drawbacks to MSC-based therapy, including its high cost, cellular phenotypic instability, and the risk of microinfarction caused by transported MSCs that become lodged in the pulmonary microvasculature [48, 49]. As a result, a new cell-free therapy with similar efficacy to that of MSCs must be developed for PNI.

Recent studies have demonstrated that the applied MSCs’ therapeutic activities are related to paracrine factors such as cytokines, proteins, and especially their EXOs [50]. Recently, EXOs have been identified as the main paracrine effectors of MSCs and can mediate cell-to-cell communication and maintain homeostatic and dynamic microenvironments for tissue regeneration [51, 52]. MSC-derived EXOs have the potential to activate PI3k/Akt, ERK, and STAT3 signaling pathways to induce the expression of several growth factors (GFs) like NGF, insulin-like growth factor-1 (IGF-1), and stromal-derived growth factor-1 (SDF-1) [53]. EXOs derived from MSCs can also activate Wnt/b-catenin and Notch signaling pathways. The Wnt signaling pathway is involved in the control of inflammation after being activated by injury [54–56]. Moreover, studies have demonstrated that exosomal miRNAs (miR-221, miR-218, miR-199b, miR-148a, and miR-135b) can promote neuronal differentiation, proliferation, and axonal outgrowth [57–59]. Also, a study has demonstrated that the miR-17-92 cluster can promote axonal outgrowth, neurogenesis, and functional recovery by activating the PI3K/protein kinase B/mechanistic target of the rapamycin/glycogen synthase kinase 3-β signaling pathway [60, 61].

In another study, Zhang et al. [62] also demonstrated that MSC-derived EXOs carry an elevated level of the miR-17-92 cluster, which can activate the PTEN/mTOR signaling pathway in recipient neuron cells. Several studies have demonstrated that MSCs with miRNA overexpression are better influenced by functional recovery in PNI situations than naive MSCs [63]. Indeed, the function of MSC-derived EXOs depends on the condition of the original cell that releases EXOs (like SC-derived EXOs), which is related to the miRNA content of EXOs and influences their biological function.

Furthermore, recent studies have shown that MSC-derived EXO can upregulate miRNA and promote angiogenesis [64]. MSC-derived EXOs are identified as the main immunomodulatory mediators due to their immunomodulatory proteins [65]. About this, several studies have demonstrated that MSC-derived EXOs have a positive immunomodulatory effect in various pathologic conditions due to their induction of high levels of anti-inflammatory cytokines like IL10 and TGF-β1 and their enhancement of the expression of IL1B, IL6, TNFA, and IL12P40 as proinflammatory factors [66]. Also, MSC-derived EXOs induce regulatory T cells (Tregs), which are recognized as immune tolerance agents [67]. The majority of these studies about the effects of MSC-derived exosomes on the nerve injuries are summarized in Table 2. Altogether, these outcomes show a superior potential role for MSC-derived EXOs and their miRNA in the regeneration of PNI (Fig. 2).

**Table 2:** Summary of the positive effects of MSCs-derived exosomes in the studies for PNI

Cell source	In vitro/In vivo	Exosome isolation methods	Exosome concentration	Signaling pathway/related exosomal cargo	Effect	References
Gingival mesenchymal stromal cells (GMSCs)	In vitro and in vivo	Differential ultracentrifugation	100 μg/mL (in vitro) 10 μL PBS containing 10 μg exosomes (in vivo)	–	The in vitro studies demonstrated the exosomes from GMSCs promote SCs proliferation and DRG axon growth. The in vivo studies demonstrated exosomes from GMSCs increase the number and diameter of nerve fibers and promote myelin formation	[80]
Human adipose-derived mesenchymal stromal cells (hAMSCs)	In vitro	Differential ultracentrifugation	100 ng/ml Exo	The protective effect of hAMSCs-derived exosomes was mediated by activating the PI3/K-Akt signaling pathway	hAMSC-derived exosomes have protective effect against neuron damage induced	[81]
GMSCs	In vitro and in vivo	Exo Quick kit	20 μg/mL (in vitro) 40 µg in 20 µl PBS (in vivo)	GMSC-derived exosomes can upregulate the expressions of c-JUN, Notch1, GFAP, and SOX2	GMSC-derived exosomes promoted proliferation and migration of Schwann cells	[82]
Bone marrow mesenchymal stromal cell (BMMSC) exosomes	In vitro	Differential ultracentrifugation	3 × 10^8^ (diluted in 300 μl of growth medium) MSC-exosomes	MSC-exosomes have a potential to activate the PTEN/mTOR signaling pathway in recipient neurons Also, miR-17–92 mediated axonal growth	BMMSCs-derived exosomes enhance axonal growth, which provides a potential therapeutic alternative to promote axonal growth	[62]
hAMSCs	In vitro and in vivo	Differential ultracentrifugation	–	The hAMSC-derived exosomes enhanced axonal regeneration due to the presence of growth factors, BDNF, FGF-1, GDNF, IGF-1 and NGF in them	hAMSC-derived exosomes are involved in peripheral nerve regeneration and have the potential to be applied as a therapeutic strategy for effective tissue-engineered nerves	[83]
hAMSCs	In vitro	Exo-spinTM kit	40 μg/mL	hAMSC-derived exosomes promote neural differentiation and inhibit cell apoptosis due to existence of growth factors, proteins and miRNAs	hAMSC-exosomes have potential to enter into the cells and promote cell growth, viability, and also induce neural differentiation of PC12 cells	[84]

## MSC therapy versus MSC-derived exosomes in clinical practice

So far, MSC therapies have emerged as a powerful tool in clinical practice for the treatment of various diseases, such as neurodegenerative disease [68]. Although MSCs can differentiate into various cells like nerve cells and also regulate the microenvironment of the injured area to accelerate the regeneration of peripheral nerves [69], a few stem cell products derived from mesenchymal stromal cells and clinical trials have applied MSCs after nerve injury due to certain disadvantages of this application [70]. The lack of clinical usage of MSCs in peripheral nerve therapies may be due to several unavoidable drawbacks with regards to MSC therapy. One of these drawbacks is that the large diameter of these cells may lead to their aggravation in the lung after intravenous injection and, thus, infusion toxicity [71]. Furthermore, MSC injections may result in oncological complications. In addition, aging is another limitation [72]. Thus, the clinical application of MSCs raises some ethical and safety concerns since they are limited to nerve regeneration [69]. Therefore, the optimal approach for the use of derivatives of MSCs to take their advantages to repair PNI can be useful.

As mentioned, some of the observed beneficial effects of MSC therapies can be partly due to their paracrine action rather than the long-term engraftment of transplanted MSCs [73]. Also, studies have investigated whether MSC-derived extracellular vesicles like EXOs exert functions similar to those of MSCs by beneficially promoting peripheral nerve regeneration [74, 75]. So it can be concluded that MSC-derived exosomes, as cellular paracrine products may play a major role in recovery post-nerve injury [74]. It is widely accepted that MSC-derived EXOs play an essential role in the amelioration of disease [76]. On the other hand, the advantages of EXO therapies are related to their safety profile [68]. The safety profile of MSC-derived EXOs therapy is a critical consideration in its clinical application [77]. Current evidence suggests that EXOs have a proper safety profile, with low immunogenicity, permeability, easy storage (they can be lyophilized), and minimal adverse effects reported in clinical trials [78]. However, the manufacturing process of EXO-based clinical products needs to be standardized to ensure consistency in quality, safety, and efficacy [79]. Consequently, MSC-derived EXOs have been subject to much research interest in recent years.

## Therapeutic and clinical application of exosomes in neurodegenerative disease

### Angiogenesis and vascular regeneration

The vascular network plays a critical role in maintaining the microenvironment homeostasis of the peripheral nervous system through the supply of oxygen and nutrients, which are essential for the regeneration of the PNS [85]. Also, several studies have demonstrated that there is an interlinkage between nerve regeneration after injury and vascularization [86]. Furthermore, the vascular networks can provide tracks for SCs to migrate along, and endothelial cells (EC) secrete various bioactive agents that are conducive to neurite elongation [86, 87]. So, reconstruction of the vascular network following PNI is another purpose for the regeneration of the peripheral nerve. However, in current strategies of treatment such as nerve guide conduits and decellularized grafts, vascular regeneration is one of the main challenges [88, 89].

In tissue regeneration, reconstructing blood vessels and blood flow in injured and ischemic tissues is necessary [90–92]. Recently, several studies have illustrated that EXO can promote vascular regeneration as a key regulator. The study of Nooshabadi et al. [64] has shown that human endometrial MSC-derived EXOs have a positive effect on the angiogenesis process in a dose-dependent manner and can be applied in the treatment of vascular disease and wound healing. Zhang et al. [93] found that umbilical cord MSC-derived EXOs can promote vascular regeneration in ECs by activating the Wnt/β-catenin pathway. Also, Liu et al*.* [94] have shown that EXOs from induced pluripotent stem cell-derived mesenchymal stem cells enhance angiogenesis due to activating the PI3K/AKT signaling pathway in EC cells. Moreover, Gong et al*.* [95] demonstrated that proangiogenic miRNAs can be transferred within ECs through generated exosomes to enhance angiogenesis. Thus, it is reasonable to hypothesize that exosomes promote angiogenesis by at least three distinct mechanisms, including:

1. EXOs can promote EC survival and proliferation by upregulating Cyclin-D1 and downregulating p53, p21, and p27.
2. EXOs promote angiogenesis in EC cells by activating signaling pathways such as Wnt/β-catenin and PI3K/AKT.
3. EXOs contain proangiogenic miRNAs that are transferred between ECs.

On the other hand, studies have shown that EXOs control vascular regeneration after PNI to change how peripheral nerve regeneration happens. A study by Xin et al. [96] showed that the MSC-derived EXOs can enhance functional recovery, neurogenesis, neurite remodeling, and angiogenesis. Similar effects were found in another study, which showed that MSC-derived EXOs can promote neurogenesis and angiogenesis and reduce inflammation in rat models [97, 98]. All of the mentioned studies about the angiogenesis effects of MSC-derived exosomes are summarized in Table 3. In summary, all of these studies demonstrated that vascular regeneration and angiogenesis that are intermediated by EXOs are conducive to peripheral nerve regeneration, which can be a superior therapeutic strategy for PNI repair by facilitating angiogenesis and vascular regeneration.

**Table 3:** Summary of the angiogenesis effects of MSCs-derived

Cell source	Exosome isolation methods	Exosome concentration	In vitro/In vivo	Signaling pathway/related exosomal cargo	References
Human umbilical cord mesenchymal stromal cells (hucMSC)	Differential centrifugation	80 and 160 mg/ml (in vitro) 200 mg suspended in 200 ml (in vivo)	In vitro and In vivo	hucMSC- exosomes promoted angiogenesis by delivering Wnt4 to activate Wnt/β-catenin in endothelial cells	[93]
MSCs line C3H10T1/2 cells	ExoQuick-TC kit	(100 μg/ml)	In vitro	MSCs-derived exosomes regulated the delivery of miRs from MSCs to recipient cells and promoted angiogenesis	[95]
Human endometrial mesenchymal stem cell	Differential centrifugation	0, 25, 50, 100, 150, and 200 µg/mL	In vitro	–	[64]
Human umbilical cord blood (UCB)	Differential centrifugation	100 μg/mL (in vitro) 200 μg dissolved in 100 μL PBS (in vivo)	In vitro and In vivo	UCB-exosomes enhance angiogenesis due to the presence of miR-21-3p that inhibited of phosphatase and tensin homolog (PTEN) and sprouty homolog 1 (SPRY1)	[99]
bone marrow mesenchymal stem cells	ExoQuick-TC	80 μg dissolved in 100 μL PBS	In vitro and In vivo	MSC-derived exosomes significantly stimulate neovascularization by delivering miRNAs	[100]
hAMSCs	ExoQuick-TC	5 μg/mL	In vitro	hAMSC-derived exosomes mediated angiogenesis due to release microRNAs (miR-132 and miR-146a) that these microRNA increased the expression of proangiogenic genes in human umbilical vein endothelial cells	[101]
Bone marrow-derived mesenchymal stromal cells	Differential centrifugation	100 ng/mL	In vitro	Exosomes regulate angiogenesis due to contain both pro- (Ang-2, ET-1, EG-VEGF/PK1, persephin, uPA) and anti-angiogenic factors (TSP-1, TIMP-1, PEDF, PAI-1)	[102]

### Axon outgrowth and regeneration

Recently, several studies have demonstrated that exosomes modulate axonal regeneration due to the transfer of specific exosome contents, such as protein, and microRNAs, from SCs to axons [103]. Also, multiple studies discussed how axonal regeneration can be promoted by EXOs, and most of these studies demonstrated that derived EXOs from various cell sources can promote axonal regeneration by impinging directly on the phosphatase and tensin homolog (PTEN), the mechanistic target of rapamycin (mTOR) signaling pathway [104, 105]. The PTEN-mTOR pathway is a key factor in axonal regeneration. Accordingly, EXOs have neurophysiological activities that can promote neurite outgrowth [104].

A previous study has demonstrated that EXOs derived from SCs can be internalized by axons and also increase axonal regeneration in in vitro and in vivo studies [32]. Mechanically, EXOs have the potential to change growth cone morphology to a pro-regenerative phenotype and can also decrease the activity of the GTPase RhoA, which is involved in axon retraction and growth cone collapse [32, 106]. Another main factor in EXOs that facilitate axon regeneration is microRNAs like miR-21. A study showed that expression of miR-21 can promote dorsal root ganglion (DRG) axon regrowth [107]. Also, miRNA can facilitate axon regeneration in peripheral nerves due to the knockout of Dicer, a key activator of the RNA-induced silencing complex (RISC) [75, 108].

The results of studies suggest that EXOs can be sent to recipient neurons as effective miRNAs to control the growth of axons. Furthermore, in another study, Buccan et al. [83] demonstrated the effect of MSC-derived EXOs on neurite outgrowth. Their results have shown that cultured dorsal root ganglia (DRG) neurons with MSC-derived EXOs increased the neurite outgrowth of the DRG neurons after co-culturing with EXOs due to the presence of growth factors like BDNF, FGF-1, GDNF, IGF-1, and NGF in the MSC-derived EXOs.

**Fig. 3:**
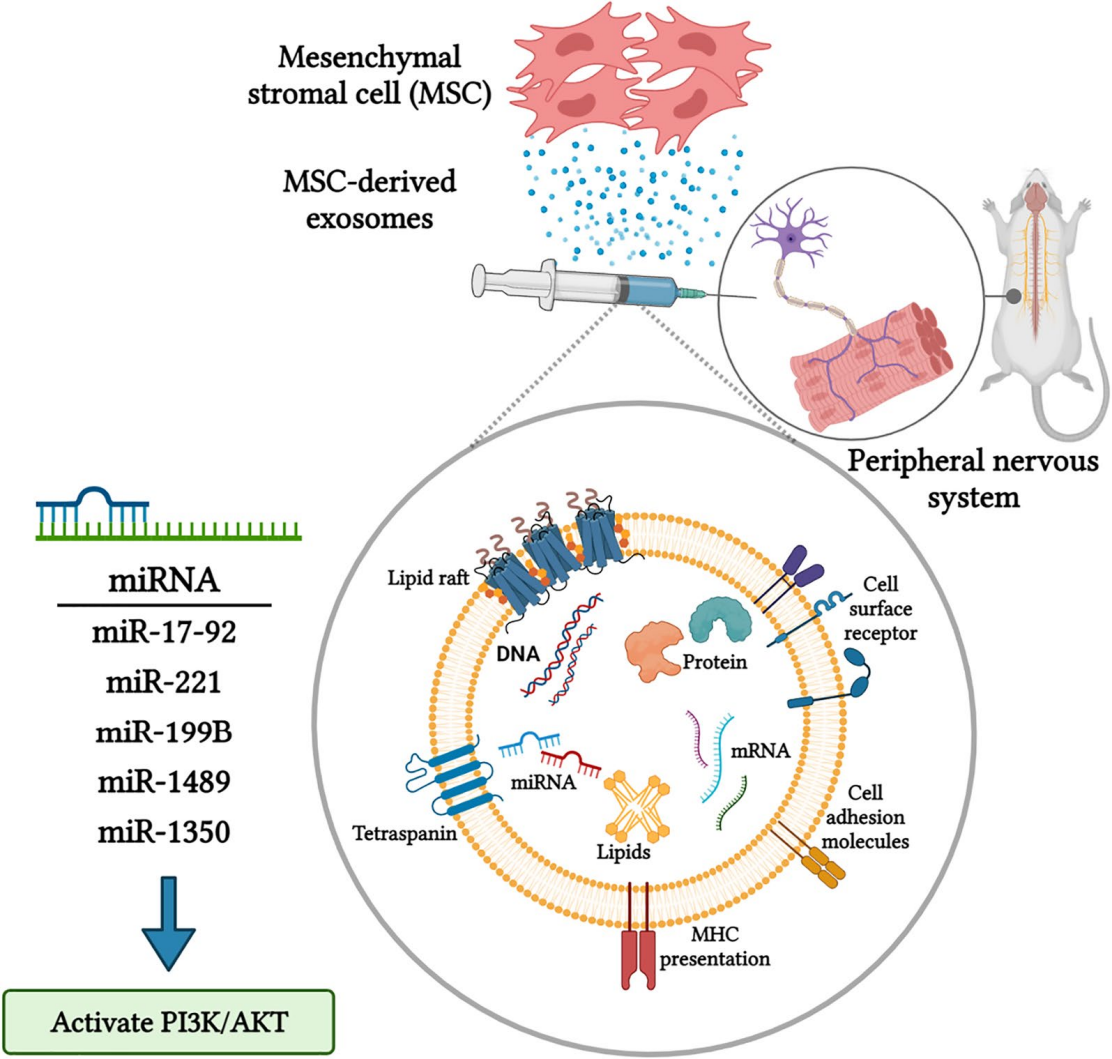
Diagram illustrates how MSC-derived exosomes can regulate expression of miRNA and activate PI3K/Akt, which induce activation of the PI3/AKT signaling pathway in neural cells, leading to promotion of nerve regeneration

A recent study by Shariati et al. [84] demonstrated that human adipose stem cell (human ADSC)-derived exosomes penetrate into the target cells and increase viability, cell growth, and induce neural differentiation of PC12 cell lines due to the existence of growth factors, proteins, and miRNAs. Overall, evidence has demonstrated that MSC-derived EXOs have the potential to promote axonal regeneration through three main mechanisms (Fig. 3):

1. Transport of miRNAs to induce axonal outgrowth
2. Shuttle neurotrophic growth factors facilitate axon regeneration.
3. Impinging directly on the PTEN-mTOR pathway

### Neuroinflammation

Neuroinflammation is one of the key factors in recovery from PNI. When PNI has occurred, myelinating SCs are dedifferentiated and activated in the distal stump of the nerve. Dedifferentiated SCs begin to clear cell debris and residual injured myelin in a Wallerian degeneration (WD) process. On the other hand, the differentiated SCs release several chemokines and proinflammatory cytokines that lead to a neuroinflammatory response [109, 110]. The neuroinflammatory response leads to the accumulation of peripheral immune cells, like circulating macrophages, at the injury site [111]. Circulating macrophages are essential for the regeneration of axons due to the clearance of axonal and myelin debris because degenerated axon debris inhibits axonal growth in the later stages of WD [112, 113].

Despite the fact that neuroinflammation has a double-edged sword effect, neuroinflammation has some positive effects in the process of regeneration from nerve injury, but excessive inflammatory responses can not only be obstacles for nerve regeneration but also cause neuropathic pain. Hence, an appropriate level of neuroinflammation is the main target for PNI.

**Fig. 4:**
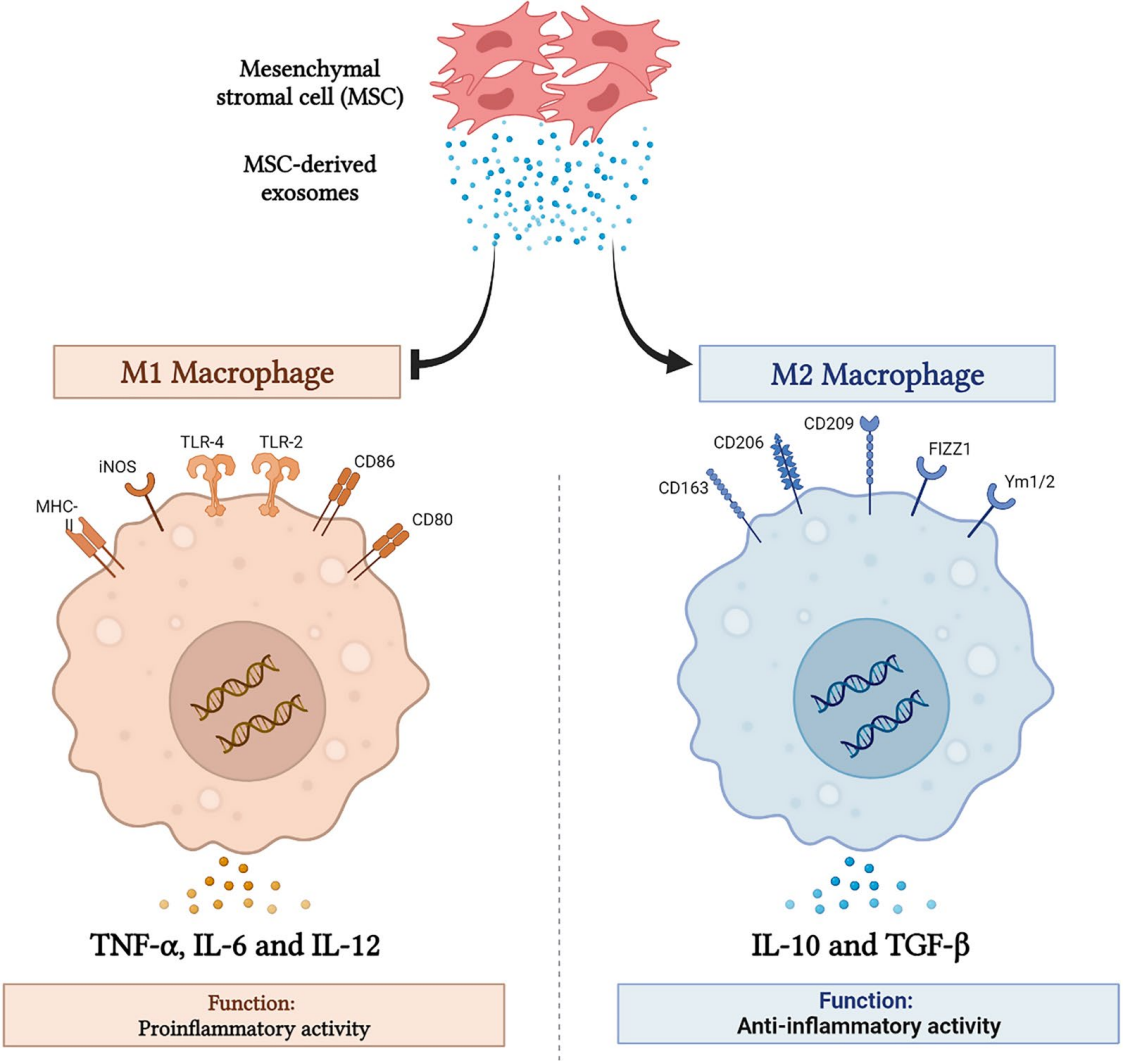
MSC-derived exosomes exhibit immunomodulatory and anti-inflammatory effects, which decrease nerve tissue damage. MSC-derived exosomes have an immunomodulatory effect due to the interaction of exosomal miRNAs. MSC-derived exosomes are able to transform macrophages from the M0 and M1 phenotypes to the M2 phenotype. Also, they increase secretion of M2-related cytokines such as TGF-β and IL-10 and also decrease M1-related cytokine levels (IL-6, IL-12, and TNF-α)

Multiple studies have reported that the main immunosuppressive effects of MSCs are related to the immunoregulatory properties of their secretome, such as EV exosomes [114, 115]. MSC-derived EXOs demonstrate their immunomodulatory effect via miRNAs. For example, a study demonstrated that miR‐21 in MSC-derived EXOs can modulate immunoreactions by diminishing signal transducers and activators of transcription 3 (STAT3) expression and inhibiting the nuclear factor kappa β (NF‐κβ) pathway [116]. Furthermore, miR‐181c, which is found in MSC-derived EXOs, plays a key role in reducing inflammation by reducing NF‐κβ activation and repressing the toll-like receptor 4 (TLR4) signaling pathway [117]. Another miRNA that exists in MSC-derived EXOs is miR‐21‐5p. It has been demonstrated that miR‐21‐5p diminished proinflammatory cytokines and increased M1 to M2 polarization in alveolar macrophages by inhibition of iNOS mRNA expression (Fig. 4) [118]. In the same way, miR‐326, miR‐182, miR‐17‐ 5p, miR‐140‐5p, miR‐9, and miR‐let7 that are found in MSC-derived EXOs can also reduce inflammation by suppressing proinflammatory cytokines [119].

Indeed, MSC-derived exosomes with specific miRNAs can induce the polarization of macrophages from the M1 to the M2 phenotype to promote nerve regeneration. In a nerve tissue injury situation, macrophages differentiate into M1 macrophages that can promote an inflammation response and also aggravate tissue damage. But MSC-derived exosomes induce the polarization of macrophages from the M1 to the M2 phenotype to promote nerve regeneration due to their specific miRNAs (Fig. 4). Collectively, we can conclude that MSC-derived EXOs modulate neuroinflammation to promote axonal outgrowth.

## Exosomes ameliorate neuropathic pain

Neuropathic pain is a type of chronic pain that occurs as a result of a lesion or disease in the peripheral nervous system [120, 121]. Although the exact mechanisms of neuropathic pain as a chronic pain are poorly determined, studies have reported that neuropathic pain is mostly related to neuroinflammation [122]. Up until now, clinical treatment strategies for neuropathic pain have included physical, pharmacological, and interventional approaches, but none of them appear to be effective in controlling the condition [123]. Therefore, an effective clinical strategy is necessary for the treatment of neuropathic pain. Nowadays, exosome therapies represent a potential candidate for clinical neuropathic pain treatment due to their anti-neuroinflammation effects [124]. Clinical studies have suggested that EXOs can modulate immune responses and promote the healing process, thereby potentially alleviating inflammation and pain [125]. Indeed, EXOs can suppress the production of proinflammatory cytokines such as TNF-α,IL-1β, and PGE2 in tissue-injured areas and also stimulate the release of IL-10, leading to antinociceptive effects [126]. Exosomal miRNAs like miR-181c-5p, miR-216a-5p, and miR-126-3p have also been demonstrated to ameliorate neuropathic pain in sciatic nerve compression in in vivo studies [123, 127]. EXOs can ameliorate neuropathic pain by reducing proinflammatory cytokines and promoting neuronal proliferation and function [128].

## Exosomes as nanocarriers

Exosomes are lipid bilayer-enclosed vesicles that originate from the internal budding of the late endosomal membrane and are secreted by all types of cells. Exosomes are therefore a natural cargo for cell–cell communication. This property attracted the attention of researchers to exosome-based gene or drug delivery systems. Exosome-based delivery systems have several advantages over other delivery systems due to the following reasons:

1. A variety of biological cargoes can be delivered by exosomes, like drugs, small RNAs, mRNAs, and proteins.
2. Natural capacity of exosomes to cross biological barriers, like the blood–brain barrier.
3. Exosomes can transfer into other tissues with no blood supply.
4. Exosomes can influence targeted tissue for a long period of time.
5. Exosomes are biocompatible and genetically engineered.
6. To avoid systemic toxicity, exosomes can be engineered as surface proteins to distinguish specific targeted tissues and avoid unwanted accumulation in surrounding tissues [129].

Exosomes transport genes or drugs by fusing with the cell membrane of the receptor in acidic environments. Indeed, studies have demonstrated that exosomes have the tetraspanin protein CD9 on their surfaces [130, 131], which can fuse with the target cell membrane to transport a specific gene or drug for therapy. Exosomes have thus been used as an effective carrier for in vivo drug or nucleic acid delivery by researchers. But several challenges have existed regarding the clinical use of exosomes as carriers, such as methods to transport drugs or nucleic acids into exosomes efficiently. Various methods have been applied to import proteins, drugs, and nucleic acids into exosomes, such as incubation, freeze–thaw cycles, sonication, extrusion, electroporation, thermal shock transfection, saponin-assisted loading, hypotonic dialysis, and the pH gradient method [132].

Although many clinical trials are being conducted to examine the therapeutic effect of exosomes in a range of clinical settings such as SARS-CoV-2 pneumonia, acute ischemic stroke, macular holes, and cerebrovascular disorders [133], only a few preclinical studies in peripheral nerve regeneration have been conducted. Yang et al. [134] showed that the implementation of nerve guidance conduits containing NT-3 mRNA-loaded ADSC-derived exosomes significantly improved gastrocnemius muscle function in a rat sciatic nerve defect model. Neurotrophin-3 (NT-3) concentration, a prominent neurotrophic factor in peripheral nerve regeneration, is not sufficient in the early phase of nerve injury. Therefore, in this study, ADSCs were transduced by NT-3 mRNA, and exosomes extracted from these cells were embedded in alginate hydrogel to build a nerve guidance conduit. Sustained release of NT-3 mRNA containing exosomes was detected at least after 2 weeks of NGC [134]. Notable nerve regeneration and functional recovery of the gastrocnemius in treated rats were observed.

Fan et al. [135] found that miR-146a-loaded MSC-derived exosomes helped improve diabetic peripheral neuropathy (DPN). Exosome injection increased both mechanical and thermal stimulus thresholds while decreasing nerve conduction velocity. miR-146a, an anti-inflammatory factor whose expression mediates dorsal root ganglion survival in DPN, was transfected into mouse bone marrow-derived mesenchymal stem cells (BMMSCs). Exosomes derived from transfected MSCs were injected once a week for four weeks to improve conduction velocity and thermal and mechanical stimulus threshold in treated groups of mice.

Singh et al*.* [136] fused BMMSC-derived exosomes with polypyrrole NPs (PpyNPs)-loaded liposomes via a 10-time freeze–thaw process. This hybrid could provide both chemical and electrical cues for nerve regeneration, as exosomes contain chemical ingredients and PpyNPs induce electrical conduction for nerve regeneration. Intramuscular injection of the hybrid notably normalized the compound muscle action potential and the nerve conduction velocity in DPN rats. Surprisingly, hyperglycemia and weight loss have been controlled in the treated group as a result of the paracrine effect of the hybrid injection.

Liu et al*.* [137] demonstrated the crosstalk between SCs and neurons in the peripheral nerve. The researchers discovered that miR-21 levels are lower in SC-derived exosomes from diabetic peripheral neuropathy rats, which may impair their ability to induce nerve regeneration. Exosomes derived from transduced SCs with miR-21-lentiviral vectors improved the cells’ ability for neurite growth induction in a high glucose condition compared with exosomes derived from glucose-exposed SCs. It has also been demonstrated that miR-21 exerts its effect partly through p-AKT signaling. The promising results encourage more investigation for exosome therapy to move forward.

## Advantages of exosomes as nanocarriers in comparison to other synthetic vesicles

Since the 1990s, more than 50 man-made nanoparticles have been approved for use in clinical settings. All of these nanoparticles are simple two-layer lipids with a few extra ingredients [138]. Even though it seems unlikely that more complex nanocarriers with more of a natural biological structure and cargo could be made on a large scale, exosomes could be used to test the potential of these kinds of multifunctional drug carriers.

Besides, applying exosomes is likely to facilitate issues associated with drug loading and delivery, which substantially reduce the efficiency of nanoparticle production. Both loading exosomes with the cargo of interest and surface modification can be obtained through natural cellular processes in exosome production as well as cargo delivery via endocytosis/membrane fusion [139]. Naturally loaded exosomes can be produced through genetically engineered cells of origin to produce the desired molecules inside and/or on the cell membrane, bypassing cargo degradation and receptor implantation during nanoparticle synthesis [138].

Although nanomedicines mostly evoke fewer side effects in comparison to free drugs due to their less frequent encounter with non-targeted tissues, some polyethylene glycol (PEG)-conjugated nanoparticles can trigger inflammatory and rarely life-threatening reactions. Early-phase clinical trials have proven allogenic MSC-derived EVs safe [140, 141]. Similarly, there is a growing body of evidence on the safety of blood-derived EVs in blood transfusions that could be used to predict the safety profile of allogenic exosomes [138, 142]. However, due to the enormously heterogeneous cargo of exosomes, which may affect off-target results, caution should be exercised in generalizing this data [143].

The mechanical stiffness of exosomes is another advantage, as shown in a study using extracellular matrix-simulating hydrogels. EVs are superior to nanocarriers in both their tolerance of a stress-relaxing environment and their ability to cross biological barriers like the blood–brain barrier due to their surface proteins [133, 144]. Therefore, the immunocompatibility and organ-organelle tropism of exosomes may serve them as more efficient therapeutic agents.

## Challenges of exosome therapy

Although the favorable results from exosome therapy in preclinical and clinical studies are encouraging, as an unprecedented therapeutic approach, there are some issues that need to be tackled before applying them in clinical settings. Firstly, the lack of GMP-compliant large-scale production techniques has hindered the transition of exosome therapy from preclinical to clinical studies. A variety of methods to propagate cell sources, from 3-D cell culture to bioreactors, have been applied, yet they demand more improvements to efficiently meet the need for a clinical dose of exosomes [145].

Secondly, in the absence of effective isolation techniques, exosomes are precipitated with other undesirable molecular contaminants, which impede the clinical translation of the therapeutic agents. Differential centrifugation followed by ultracentrifugation has been more frequently applied in preclinical studies [146] than the other currently available methods, including size-exclusion chromatography (SEC) density, gradient ultracentrifugation, precipitation, immunoaffinity-based capture [147], and microfluidics-based technologies. However, while the technique has proven effective, coprecipitation of non-exosome molecules, low efficiency, and impaired exosome structure remain issues to be settled [148].

Thirdly, by carrying a diverse range of molecules with synergic or additive effects, EVs may intensify the therapeutic effects, yet some concerns remain due to their potential oncogenic activity, particularly for those driven by stem cell proteins [149, 150]. In other words, the inability to precisely both characterize and quantify exosomes’ cargo as well as the inability to target them for a specific receptor raises off-target concerns.

Fourthly, the lack of techniques for precise quantification of exosomes poses pharmacokinetic challenges. Available methods of exosome measurement are highly sensitive and accurate and include nanoparticle tracking analysis, electron microscopy, surface plasmon resonance, flow cytometry, dynamic light scattering, tunable resistive pulse sensing, and single-particle reflectance imaging sensors. However, these techniques are quite costly and laborious, particularly when it comes to large-scale production.

Fifthly, optical imaging demonstrated exosomes’ rapid accumulation in the liver and spleen following intravenous injection, which represents their undesirable biodistribution and short half-life. The considerably lower half-life of exosomes than nanocarriers (60 min vs. several hours) is another shortcoming that needs to be overcome [149]. Therefore, a range of issues should be avoided, from the manufacturing of exosomes to accurately characterizing and quantifying them, in order to guarantee a safe and effective therapy with known possible side effects. Hence, the International Society for extracellular vesicles (ISEV), established in 2014, updated isolation and characterization methods in 2018 to further accurate and reliable EV isolation [151]. Furthermore, the EV-TRACK platform, developed in 2017, encourages authors to share their isolation and characterization techniques and receive advice on possible drawbacks to enhance more reproducible and concrete results [133].

## Future perspectives and conclusions

When a peripheral nerve injury occurs, a series of complex events occur in the neuron’s cell body and in surrounding cells. Several factors are involved in the nerve regeneration process, such as inflammation, trophic factors, angiogenesis, and SCs. Due to the requirement to sacrifice a healthy tissue nerve, current Schwann cell-based therapy to regenerate peripheral nerves is not an ideal approach. Furthermore, the use of autologous SC exosomes to treat PNI does not overcome the obstacle of needing to sacrifice a functioning nerve to gain the SC-derived exosomes. On the other hand, MSCs have been shown to be efficacious in improving neurite outgrowth, and they are applied in PNI studies. MSC transplantation using nerve guide conduits has shown positive effects in animal models of nerve gap injuries but is still far from being widely accepted.

Recently, MSC-derived exosomes have been known as the main regulatory mediator that mediates tissue regeneration. MSC-derived exosomes have a therapeutic effect similar to MSCs and, due to several advantages over MSCs, can be used as a cell-free therapy to treat peripheral nerve injury instead of MSCs. MSC-derived exosomes play a pivotal role in mediating intercellular communication in the peripheral nerve microenvironment. Indeed, MSC-derived exosomes transfer genetic substrates such as miRNAs, neurotrophic factors, and proteins to axons to regulate axonal regrowth, as described in this study. Moreover, MSC exosome-based therapy can resolve the issues caused by stem cell transplantation. Hence, in the future, MSC exosome-based therapy will be a cell-free approach for regenerating PNI. Although several studies have shown that injecting MSC-derived exosomes into nerve stumps or supplementing nerve conduits for the treatment of peripheral nerve injury is effective and safe, further research is needed to determine the potential of MSC-derived exosomes for clinical application.

## Data Availability

The data that support the findings of this study are available from the corresponding author upon reasonable request.

## Abbreviations

PNI: Peripheral nerve injury
SCs: Schwann cell
MSCs: Mesenchymal stem cells
NGCs: Nerve guide conduits
GFs: Growth factors
MVs: Microvesicles
EXOs: Exosomes
EV: Extracellular vesicles
WD: Wallerian degeneration
PTEN: Phosphatase and tensin homolog
IGF-1: Insulin-like growth factor-1
Tregs: Regulatory T cells
mTOR: Mechanistic target of rapamycin
DRG: Dorsal root ganglion
RISC: RNA-induced silencing complex
NT-3: Neurotrophin-3
PpyNPs: Polypyrrole NPs
DPN: Diabetic peripheral neuropathy
SEC: Size-exclusion chromatography
ISEV: International Society for Extracellular Vesicles
